# Role of Calcium-Activated Potassium Channels in Proliferation, Migration and Invasion of Human Chronic Myeloid Leukemia K562 Cells

**DOI:** 10.3390/membranes13060583

**Published:** 2023-06-04

**Authors:** Valeria Y. Vasileva, Zuleikha M. Khairullina, Anastasia V. Sudarikova, Vladislav I. Chubinskiy-Nadezhdin

**Affiliations:** Institute of Cytology, Russian Academy of Sciences, Tikhoretsky Ave. 4, 194064 Saint-Petersburg, Russia; vasileva.valeriia@gmail.com (V.Y.V.); khairyllinaa@mail.ru (Z.M.K.); anastasia.sudarikova@gmail.com (A.V.S.)

**Keywords:** calcium-activated potassium channels, SK channels, IK channels, human chronic leukemia K562 cells, cell migration, cancer cell invasion

## Abstract

Calcium-activated potassium channels (KCa) are important participants in calcium signaling pathways due to their ability to be activated by an increase in intracellular free calcium concentration. KCa channels are involved in the regulation of cellular processes in both normal and pathophysiological conditions, including oncotransformation. Previously, using patch-clamp, we registered the KCa currents in the plasma membrane of human chronic myeloid leukemia K562 cells, whose activity was controlled by local Ca^2+^ entry via mechanosensitive calcium-permeable channels. Here, we performed the molecular and functional identification of KCa channels and have uncovered their role in the proliferation, migration and invasion of K562 cells. Using a combined approach, we identified the functional activity of SK2, SK3 and IK channels in the plasma membrane of the cells. Selective SK and IK channel inhibitors, apamin and TRAM-34, respectively, reduced the proliferative, migratory and invasive capabilities of human myeloid leukemia cells. At the same time, the viability of K562 cells was not affected by KCa channel inhibitors. Ca^2+^ imaging showed that both SK and IK channel inhibitors affect Ca^2+^ entry and this could underlie the observed suppression of pathophysiological reactions of K562 cells. Our data imply that SK/IK channel inhibitors could be used to slow down the proliferation and spreading of chronic myeloid leukemia K562 cells that express functionally active KCa channels in the plasma membrane.

## 1. Introduction

Ion channels are transmembrane proteins that are involved in the regulation of many physiological functions of living cells, in health and during disease. Different types of cells and tissues are characterized by various repertoires of specific ion channels that can define cellular reactions and signaling events. It is hypothesized that the migration and proliferation of cancer cells (including blood malignancies) could be critically dependent on the membrane potential and mechanical properties of the plasma membrane, as well as cellular volume changes [1,2]. The control of the above functions of malignant cells is closely related to the activity of various types of ion channels. 

Calcium-activated potassium channels (KCa channels) are a unique class among other K^+^ channels that are stimulated by the increase in intracellular Ca^2+^ concentration ([Ca^2+^]_i_). They are divided into three subgroups based on their single-channel properties: big (BK), intermediate (IK) and small (SK) conductance. Among them, BK channels are voltage-dependent and Ca^2+^-sensitive, whereas SK and IK channels are voltage-independent and are gated solely by Ca^2+^ that binds to channel-associated calmodulin. Small- and intermediate-conductance KCa channels (KCa2/SK and KCa3.1/IK channels), which are gated by sub-micromolar Ca^2+^ concentrations, are involved in the regulation of cell volume and mediate Ca^2+^-induced membrane hyperpolarization [3,4,5]. These channels are encoded by four *KCNN* genes: three subtypes for SK channels, *KCNN1–3* for KCa2.1–2.3 (SK1–SK3), and *KCNN4* for KCa3.1 (IK or Gardos channels) [4]. Importantly, the SK and IK channel activities can be distinguished by highly selective inhibitors. Moreover, the activity of the members of the SK family can also be separated from each other by their different sensitivities to apamin, a selective SK channel blocker naturally present in bee venom [4]. Particularly, SK2 channels are the most sensitive to apamin, with a half maximal blocking concentration (IC50: 27–140 pM), while SK1 channels are less sensitive (IC50: 0.7–12 nM) and SK3 channels show an intermediate sensitivity to apamin (IC50: 0.6–4 nM). Importantly, apamin has no inhibitory effects on other types of K^+^ channels nor on ion channels from other families, as was confirmed recently for a broad repertoire of channel proteins [6]. For functional identification of IK activity, TRAM-34, the analog of clotrimazole, a potent and selective blocker of IK (KCa3.1), is widely used [7,8]. 

The involvement of SK and IK channels in the regulation of various processes in blood cells was investigated. Moreover, activation of the KCa current was demonstrated for the first time in red blood cells [9]. In T lymphocytes, SK channels have been shown to be involved in the maintenance of Ca^2+^ signaling [10,11]. In human leukemic cell lines CEM and MOLT-3 as well as healthy T cells, the expression of only IK channels was found, while apamin-sensitive SK2 currents were predominant in Jurkat leukemic cells [12]. IK (Gardos) channels regulate erythrocyte volume by the transfer of Ca^2+^ uptake into K^+^ and loss of water; Ca^2+^-dependent activation of IK and subsequent K^+^ efflux cause erythrocyte dehydration (cell shrinkage) [13]. Impairment of K^+^ transport and fluid flow associated with mutations in IK channels may lead to erythrocyte disorders such as hereditary xerocytosis or stomatocytosis [14]. 

The local deformation of the red blood cell (RBC) membrane (induced by the application of a negative pressure pulse during patch formation) may activate Ca^2+^-dependent K^+^ currents due to mechano-dependent Ca^2+^ entry [15]. These experiments suggest that such mechanisms could play an important role in controlling the shape and volume of RBC. Accordingly, mechanosensitive Ca^2+^-permeable Piezo1 channels have been shown to be implicated in RBC volume homeostasis by inducing Ca^2+^ entry, which, in turn, activates IK channels and triggers erythrocyte dehydration [16]. Importantly, in our previous studies on human myeloid leukemia K562 cell line, which is of the chronic erythroleukemia type, the activity of mechanosensitive Ca^2+^-permeable (MSCa) ion channels with biophysical properties close to those of the Piezo family was discovered [17,18,19]. Moreover, coupled activation of MSCa and KCa channels was found in K562 cells [20], indicating the presence of functional complexes of Ca^2+^-permeable and KCa channels in the plasma membrane. It was shown that such complexes could be formed by the Ca^2+^ channels and KCa channels of various subtypes and could contribute to cancer-associated functions of the cells [21]. The purposes of this work are to reveal the molecular identity of KCa channels in human chronic leukemia K562 cells and to identify their possible contribution to K^+^ currents and the regulation of pathophysiological functions of the cells.

## 2. Materials and Methods

### 2.1. Cell Culture and Reagents

Human chronic myeloid leukemia (CML) K562 cells were obtained from the shared research facility “Vertebrate cell culture collection” (supported by the Ministry of Science and Higher Education of the Russian Federation, Agreement № 075-15-2021-683) of the Institute of Cytology (St. Petersburg, Russia). Cells were tested for mycoplasma, fungal and bacterial contamination in the shared research facility. Cells were grown in full RPMI-1640 medium, containing 10% fetal bovine serum (FBS, Biolot, St. Petersburg, Russia) and 80 mg/mL antibiotic gentamicin, in a humidified incubator at 37 °C and 5% CO_2_. Before the electrophysiological and immunocytochemical experiments, the cells were plated on poly-DL-lysine-coated (Sigma-Aldrich, St. Louis, MO, USA, Cat. no. P4158) glass coverslips of 0.4 × 0.4 cm or 1.2 × 1.2 cm. Selective SK blocker (apamin, Santa Cruz Biotechnology, California, CA, USA) and IK blocker (TRAM-34, Tocris, Bristol, UK) were used for the inhibitory analysis in all experiments. Tetraethylammonium bromide (TEA-Br) was purchased from Santa Cruz Biotechnology (Dallas, TX, USA). Stock solutions of apamin and TEA were prepared by dilution in distilled water and the stock solution of TRAM-34 was prepared using DMSO (Sigma-Aldrich, St. Louis, MO, USA). Working solutions were prepared prior to the experiments. 

### 2.2. Electrophysiology

The patch–clamp setup was based on the Axopatch 200B operational amplifier and Axon Digidata 1550A (Molecular Devices LLC, San Jose, CA, USA) analog-to-digital converter, controlled by a Windows-based personal computer with Axon PClamp 10.7 Software Suite (Molecular Devices LLC, San Jose, CA, USA) for data acquisition, filtration, processing and analysis. Pipettes were pulled from borosilicate glass capillaries with filament (BF-150-86-10, Sutter Instruments, Novato, CA, USA) to a resistance of ~5–10 MΩ when filled with a cytosol-like solution (see Section 2.3). All experiments were performed at room temperature (22–23 °C). Whole-cell currents were recorded using a whole-cell configuration of the patch–clamp technique, according to the following protocol: current traces were obtained from voltage steps from +20 to +80 mV in 10 mV increments (holding potential was 0 mV); the sampling interval was 1.75 s.

### 2.3. Solutions

For whole-cell experiments, micropipettes were filled with a cytosol-like solution containing (in mM): 140 KAspartate, 5 NaCl, 2 EGTA/KOH,1 MgCl_2_ and 20 HEPES/TrisOH, with the necessary amount of CaCl_2_ to establish free ionized calcium concentration [Ca^2+^]_i_ at 1 μM (pCa6). The experimental chamber solution (extracellular) contained (in mM): 145 NaCl, 2 CaCl_2_, 1 MgCl_2_ and 10 HEPES/TrisOH. The pH of all solutions was maintained at 7.3.

### 2.4. Reverse Transcription Polymerase Chain Reaction (RT-PCR)

Total RNA was isolated using the RNeasy Mini Kit (Qiagen, Germantown, MD, USA) according to the manufacturer’s instructions. The synthesis of the first-strand cDNA was performed using a commercial kit (OT-1, Syntol, Moscow, Russia). The reverse transcription reaction was carried out by mixing 1 µg of RNA with 0.5 µg random hexaprimers, 100 units of reverse transcriptase (revertase) MMLV and 2.5× of the reaction buffer in a total volume of 25 µL, followed by incubation at 37 °C for 1 h. For the negative control (RT-), a PCR without MMLV revertase was used. The concentrated cDNA was diluted to a working concentration of 100 ng/mL. The PCR primers were designed using the GeneRunner v5.0.59 software. The primer sequences for amplification of the gene of interest and expected reaction product sizes (bp) are presented in Table 1. PCR was performed in a volume of 15 µL using 1.5 µL diluted cDNA, 0.3 µM each primer, 200 µM dNTP, 2 mM MgCl_2_, 1 unit Hot-Taq polymerase and 1× Hot-Taq polymerase buffer (Syntol, Moscow, Russia). PCR was performed according to the following scheme: 9 min 30 s at 94 °C; 35 cycles of 40 s at 94 °C, 30 s at 60 °C and 30 s at 72 °C; 5 min at 72 °C. To determine the presence of a gene product, 10 μL of the PCR was subjected to electrophoresis in 6% polyacrylamide gel. The gel was stained with the DNA-binding dye GelRed (1:10,000, Biotium, Fremont, CA, USA). The results of the reaction were visualized in UV light using the E-Gel Imager System (Thermo Fisher Scientific, Waltham, MA, USA).

### 2.5. Immunofluorescence

For immunofluorescent staining, specific primary antibodies against the intracellular loop of SK2 (Alomone Labs, Jerusalem, Israel, cat. no. #APC-028), SK3 (Alomone Labs, Jerusalem, Israel, cat. no. #APC-025) channels and the extracellular loop of IK channels (conjugated to ATTO Fluor 488 dye, Alomone Labs, Jerusalem, Israel, cat. no. #ALM-051-AG) were used. Cell fixation was performed in 3.7% paraformaldehyde for 10 min at room temperature (RT), then the cells were permeabilized with 0.25% Tween-20 (only before the antibodies against intracellular epitopes (SK2 and SK3), for 10 min at RT). Nonspecific binding of the antibodies was blocked by incubating the samples in phosphate-buffered saline (PBS) containing 10% goat serum for 1 h at RT. Then, cells were incubated with SK2-, SK3- or IK-targeting primary antibodies overnight at +4 °C. Staining with fluorescently labeled secondary antibodies (Goat anti-rabbit conjugated to Cy3, Santa Cruz, Dallas, TX, USA) was performed for detection of SK2 and SK3 channels for 40 min at RT in the dark. Cell nuclei were counterstained with 4′,6-diamidino-2-phenylindole (DAPI, Sigma-Aldrich, St. Louis, MO, USA, 0.05 μg/mL, 30 min at RT). The specificity of the antibodies used was determined by eliminating the primary antibodies, followed by incubation of the cells with only secondary antibodies (fluorescent signal was not observed under these conditions). Visualization of the fluorescently labeled preparations was performed on Olympus FV3000 (Olympus, Shinjuku, Tokyo, Japan) confocal microscope. Images were analyzed in ImageJ software (NIH, Bethesda, MD, USA).

### 2.6. Cell Proliferation and Viability Assays

Cells were seeded in 4-well plates at a concentration of 5 × 10^5^ cells per well and incubated with the reagents (apamin, TRAM-34 or TEA) for 48 h. Cell counting was performed at 24 and 48 h time points using the Countess II Automated Cell Counter (Thermo Fisher, Waltham, MA, USA). To assess cell viability, a Trypan Blue solution (0.4%, Thermo Fisher Scientific, Waltham, MA, USA) was added and the number of trypan-positive cells was calculated for each experimental condition. 

### 2.7. Transwell Migration and Invasion Assays

Cell migration and invasion assays were assessed using Transwell inserts (Corning Inc, NY, NY, USA) with semi-permeable microporous membrane (10 µm thickness, 8 µm pore diameter). Before the experiments, the inserts were placed into 4-well plates (Jet Biofil, Guangzhou, China). For invasion experiments, transwells were pre-coated with 100 µL of Matrigel (1:40 dilution, 30 min at 37 °C, Corning Inc, NY, NY, USA). A serum gradient (10% FBS in the lower well) was used to induce K562 cell migration or invasion. Cells were resuspended in RPMI culture media without FBS. A total volume of 400 µL, containing 5 × 10^5^ cells, was placed in the upper well of the insert and 750 µL of full RPMI medium was added to the lower well. Cell culture plates were transferred to the incubator (5% CO_2_, 37 °C) for 4 h. Selective channel inhibitors were added to the upper and lower wells of the transwell system. The number of migrated or invaded cells was counted using Countess II Automated Cell Counter. The number of cells for each experimental condition was normalized to the mean number of cells in the control. 

### 2.8. Ca^2+^ Imaging

One hour before the experiments, K562 cells were plated on poly-DL-lysine-coated glass coverslips of 0.4 × 0.4 cm. Cells were loaded with 5 µM Fluo8-AM calcium probe (Cat. No. 21080, AAT Bioquest Inc., Pleasanton, CA, USA) for 45 min at 37 °C and 5% CO_2_ in the “2 mM Ca^2+^” solution containing (in mM): 150 NaCl, 5 KCl, 1 MgCl_2_, 2 CaCl_2_ and 10 HEPES/TrisOH, at pH = 7.3. Then, the cells were incubated in 2 mM Ca^2+^ without the dye to allow complete de-esterification of AM groups (15 min, at 37 °C and 5% CO_2_). To synchronize the cell population in the status of endoplasmic reticulum (ER) Ca^2+^ stores, the cells were pre-incubated with 1 µM thapsigargin (TG, Cat No T9033, Sigma-Aldrich, St. Louis, MO, USA) for 5 min in a “Ca^2+^-free” (0 Ca^2+^) medium containing (in mM): 150 NaCl, 5 KCl, 1 MgCl_2_, 0.2 EGTA and 10 HEPES/Tris to deplete ER Ca^2+^ stores. The time of pre-incubation was selected based on Ca^2+^ imaging experiments where TG was added to the cells under “Ca^2+^-free” conditions and the increase in fluorescent signals that indicate the rise in Ca^2+^ due to its release from the intracellular stores was registered (see Supplementary Figure S2). It could be seen that the reliable depletion of Ca^2+^ stores occurs within 2–3 min after the addition of TG. After the pre-incubation of the cells with TG, the coverslips were transferred to the experimental chamber filled with “Ca^2+^-free” solution. A micromed I LUM (Micromed, St. Petersburg, Russia) inverted luminescent microscope equipped with 20× objective, an appropriate filter set for Fluo8-AM (excitation 410–490 nm, dichroic 505 nm, 515 nm blocking filter) and a CCD camera (UHCCD01400KPB, Touptek Photonics, Hangzhou, China), controlled via ToupView software (Touptek, China), was used for the registration of the fluorescence. In the experiment, a 0 Ca^2+^ solution was rapidly changed to 2 mM Ca^2+^ (with or without the channel inhibitors) to induce Ca^2+^ entry to the cytosol. Each of the experiments was repeated independently at least 3 times. The recorded image stacks were processed in FIJI software (NIH, Bethesda, MD, USA) for the calculation of fluorescence intensity. The fluorescence intensity in 0 Ca^2+^ (F_0_, before application of 2 mM Ca^2+^) was used for the normalization of cell responses at each timepoint (F/F_0_); the data are presented as mean F/F_0_ (±SEM).

### 2.9. Statistics

The statistical analysis was performed using GraphPad Prism 8.0 software (GraphPad Software, Boston, MA, USA). A Kruskal–Wallis ANOVA with Dunn’s multiple comparisons test was used to compare mean currents at the membrane potentials in control and after application of KCa blockers. One-way ANOVA with multiple comparisons (to control values) was used to compare the number of cells in the presence of channel inhibitors with control conditions in proliferation/viability/migration and invasion assays. The effects of different concentrations of the reagent were compared using one-way ANOVA using Dunnett’s corrections with multiple comparisons of the mean values between each concentration tested and to control conditions. The comparison of the Ca^2+^ responses of the cells in the presence of apamin or TRAM-34 with control was performed using a two-tailed Student’s *t*-test. *p* < 0.05 was considered significant.

## 3. Results

### 3.1. Selective SK and IK Channel Blockers Inhibit Whole-Cell Currents in the Plasma Membrane of Human Myeloid Leukemia Cells

The first series of experiments were carried out to determine the level of functional expression of KCa channels in the plasma membrane of human leukemia K562 cells. Based on previously reported single-channel conductance (about 10 pS in a near-physiological Na-based bath solution [20]), the KCa currents could be mediated by SK or IK channels. Thus, we designed whole-cell patch–clamp experiments in which we were able to distinguish between SK and IK channels using highly specific inhibitory analysis. As selective blockers of SK and IK channels, 300 nM apamin and 1 μM TRAM-34 were used, respectively. Both pore blockers act from the extracellular side of the membrane [22,23,24]; thus, we added them to the extracellular medium over the course of sequential changes of bath solution during the recording. It is known that SK and IK channels are activated by cytoplasmic Ca^2+^ (from about 0.1 μM or more) via a calmodulin-binding mechanism [25,26]. Thus, we used the intra-pipette solution containing a high concentration of intracellular free Ca^2+^ (10^−6^ M = 1 μM or pCa6, see Section 2) for reliable activation of both SK and IK currents. The K^+^ outward current in the plasma membrane of K562 cells was recorded at positive membrane potentials in the range of +20 to +80 mV. 

**Figure 1:**
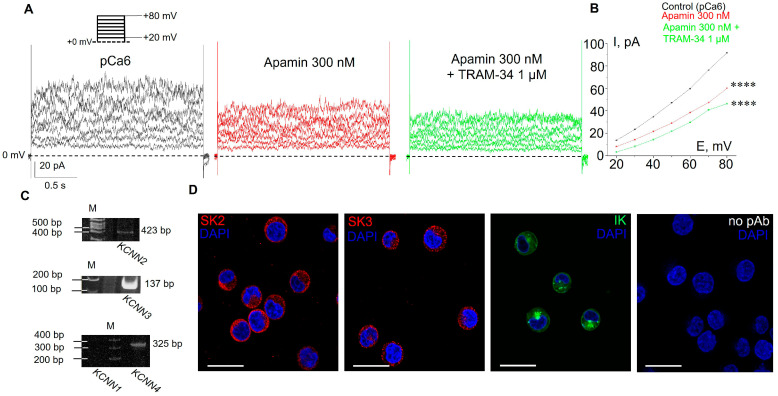
Ca^2+^-dependent potassium (KCa) channel expression and current-recording in K562 cells. (**A**) Representative whole-cell currents in the control conditions (pCa6) and after subsequent addition of apamin (300 nM, selective inhibitor of SK channels) and TRAM-34 (1 μM, inhibitor of IK channels), recorded in the range of holding membrane potentials from +20 mV to +80 mV, in 10 mV increments. (**B**) The mean (± SEM) whole-cell currents decreased after the subsequent addition of apamin and TRAM-34. Note that SE are within the size of the symbol. ****—significantly different for each condition at all membrane potentials, *p* < 0.0001. (**C**) RT-PCR analysis of KCa channel mRNA expression: M—size marker, *KCNN2* (SK2)—423 bp, *KCNN3* (SK3)—134 bp, *KCNN4* (IK)—325 bp; *KCNN1* (SK1, 282 bp) was not detected. Cropped gels are shown with enhanced contrast. Original gels are shown in Supplementary Figure S2. (**D**) Immunofluorescent staining confirmed the presence of SK2, SK3 and IK proteins in K562 cells. Blue channel—cell nuclei, red channel—SK2 and SK3, green channel—IK. No staining was observed when cells were incubated with only secondary fluorescent antibodies. Scale bar: 30 μM.

After establishing the whole-cell configuration, we first recorded outward K^+^ currents under control conditions (145 mM NaCl in bath solution; KAspartate-based pipette solution, pCa6, Figure 1). After that, we perfused the cells with apamin-containing buffer and then with both apamin and TRAM-34, to avoid the possibility of re-activation of SK channels by the washout of apamin. In whole-cell experiments, we observed a significant stepward decrease in ion currents in response to the subsequent addition of the inhibitors (Figure 1A,B), indicating the presence of both SK- and IK-mediated K^+^ channel activity in the plasma membrane. Particularly, the application of 300 nM apamin (which is sufficient to block all SK subtypes) resulted in a significant reduction of K^+^ currents, revealing SK-mediated components. The addition of 1 μM TRAM-34 (together with 300 nM apamin) resulted in further decrease in outward currents (Figure 1B), which demonstrates the contribution of IK channels. The application of TEA, a non-selective blocker of K^+^ channels of various types, further decreased outward currents (Supplementary Figure S1A). To confirm the activation of apamin- and TRAM-34-sensitive KCa currents by pCa6, we applied the same protocol in the experiments, where intra-pipette solution contained a low intracellular free Ca^2+^ concentration (10 nM, pCa8). Under these conditions, the outward currents were lower than in pCa6 and no effects of apamin or TRAM-34 application were observed (Supplementary Figure S1B). Thus, our whole-cell patch–clamp experiments using selective inhibitors unequivocally demonstrated the functional activity of both SK and IK channels in the plasma membrane of K562 cells. 

### 3.2. Functional Expression of SK2, SK3 and IK Channels in K562 Cells

As a next step, using RT-PCR analysis, we identified what subtypes of SK channels (SK1, SK2, or SK3) are expressed in K562 cells and showed the presence of mRNA of IK channel. Particularly, we detected mRNA for SK2 (KCa2.2, encoded by the *KCNN2* gene), SK3 (KCa2.3, encoded by the *KCNN3* gene) and IK (KCa3.1, encoded by the *KCNN4* gene), whereas SK1 was not detected by the reaction (Figure 1C). The immunofluorescent staining of SK2, SK3 and IK with specific antibodies confirmed the presence of the channel proteins in K562 cells (Figure 1D).

**Figure 2:**
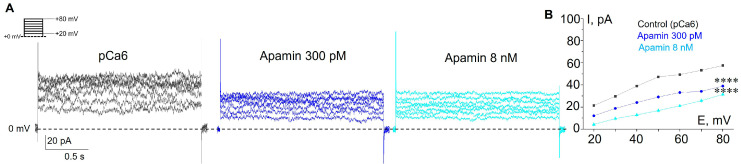
Pharmacological separation of SK2 and SK3 activity in the plasma membrane of K562 cells. (**A**) Representative whole-cell currents recorded in control conditions (pCa 6) and in the presence of 300 pM (blocks SK2 currents) or 8 nM (blocks SK3 currents) of apamin. (**B**) The mean (±SEM) whole-cell currents in control and after the addition of apamin (300 pM and 8 nM). Note that SE are within the size of the symbol. ****—significantly different for each condition at all membrane potentials, *p* < 0.0001.

Based on the results of RT-PCR, which allowed us to detect the presence of mRNA of SK2 and SK3 channels, we decided to show the impact of each of the channels on whole-cell K^+^ currents. This could be carried out because of the different levels of SK channel sensitivity to apamin: the SK2 channels are sensitive to picomolar (pM) concentrations (IC50 = 27–140 pM), whereas the SK3 channels are sensitive in the nanomolar (nM) range (IC50 = 0.6–4 nM, [4]). In whole-cell experiments, we sequentially applied two apamin concentrations: 300 pM to reliably block SK2 and 8 nM for SK3 inhibition (Figure 2). Consistently, we observed a decrease in whole-cell currents in response to both concentrations of apamin, which confirms the functional activity of SK2 and SK3 in the plasma membrane of K562 cells (Figure 2). Taken together, our electrophysiological assay evidenced the presence of functionally active SK2 and SK3 in human myeloid leukemia cells. 

### 3.3. The Effects of Selective SK and IK Channel Inhibitors on Cell Proliferation and Viability

**Figure 3:**
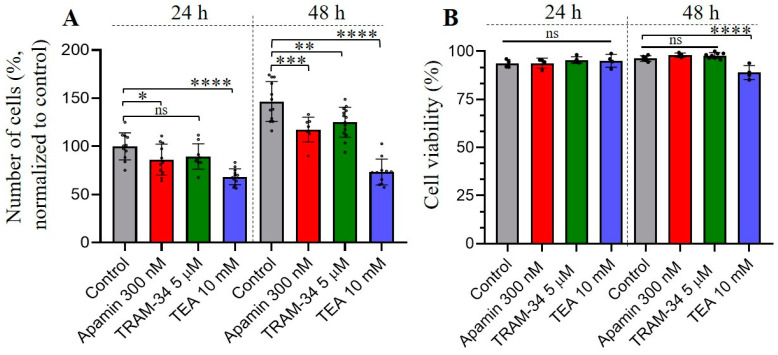
Effects of KCa inhibitors on the proliferation and viability of leukemia K562 cells. (**A**) The bar graph represents the changes in the number of cells after 24 h and 48 h from the addition of selective KCa inhibitors. A total of 500,000 cells were seeded at the starting point (0 h). Tetraethylammonium (TEA) was used as a non-selective blocker of K^+^ channels. The mean number of cells ± SD in each experimental condition (n = 8–14) are shown. * (*p* < 0.05), ** (*p* < 0.01), *** (*p* < 0.001), **** (*p* < 0.0001); all designate significantly different compared to control, using Student’s *t*-test. (**B**) No significant changes (ns) in the viability of cells were observed in the presence of selective KCa inhibitors. Note the slight decrease (88.9% viable with TEA vs. 96.3% in control) in K562 viability by TEA after 48 h. Shown is the mean percentage ± SD (n = 4–6). **** (*p* < 0.0001) significantly different, one-way ANOVA with Dunnett’s multiple comparisons. The exact *p*-values are summarized in Supplementary Table S1.

It is known that Ca^2+^ plays a crucial role in tumorigenesis, such as proliferation, migration, invasion, etc., of malignant cells [27,28]. As components of Ca^2+^ signaling pathways, KCa channels are activated by the rise in [Ca^2+^]_i_ and have been found in various cancer cell types where they can control several pathophysiological reactions [21,27]. The increase in SK3 channel expression is an unfavorable prognostic marker in renal cancer (The Human Protein Atlas, https://www.proteinatlas.org/ENSG00000143603-KCNN3/pathology, accessed on 27 April 2023), whereas IK channel expression is a poor prognosis marker for urothelial, renal, glioma and pancreatic cancer (https://www.proteinatlas.org/ENSG00000104783-KCNN4/pathology, accessed on 27 April 2023). We hypothesized that identified SK and IK channels could possibly be involved in the proliferation, cell viability, migration and invasion of human leukemia cells. Firstly, we assessed the proliferation of K562 cells under control conditions (full media) and in the presence of KCa blockers (apamin or TRAM-34) in culture media. As an additional control, the culture medium containing tetraethylammonium bromide (TEA, 10 mM), which is widely used as a non-selective blocker of K^+^ channels from various families [29], was used to test the impact of K^+^ currents on the proliferative activity of K562 cells. We observed a significant decrease in proliferation in the presence of 300 nM apamin and 10 mM TEA after 24 h incubation of K562 cells with channel inhibitors, whereas TRAM-34 also had a tendency to decrease of proliferation, but the effect was not statistically significant (*p* = 0.084) at this time point. Further incubation of the cells with specific blockers (for 48 h) allowed detection of a significant reduction in the number of cells under all experimental conditions, as compared to the control (Figure 3A). The blocking of K^+^ currents by TEA had a most prominent effect on cell proliferation, which was almost completely inhibited as compared to control (see Figure 3). Next, we addressed whether the observed effects of SK/IK blockers or TEA on cell proliferation could be due to a decrease in cell viability. Our experiments demonstrated that there were no statistically significant decreases in the number of viable cells in 300 nM apamin- or 5 μM TRAM-34-treated experimental groups, even after 48 h of incubation. At the same time, 10 mM TEA slightly—but significantly differently (as compared to control)—influenced K562 cell viability after 48 h (Figure 3B). 

### 3.4. Role of SK and IK Channels in Migration and Invasion of Chronic Myeloid Leukemia K562 Cells

**Figure 4:**
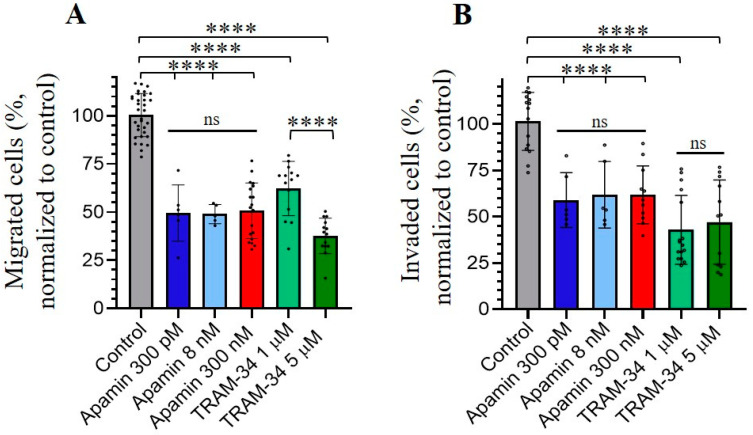
Effects of KCa inhibitors on the migration (**A**) and invasion (**B**) of leukemia K562 cells. The selective inhibitors were added to the culture media in upper and lower chambers. The graphs summarize the results of independent assays; each of the values within each assay were normalized to respective control values. The percentage of migrated (**A**) and invaded (**B**) cells is presented as means ± SD and all datapoints are shown on the graph. ns—not significant, **** (*p* < 0.0001) significantly different, one-way ANOVA test.

In our last experiments, we aimed to elucidate if SK and IK channels may also be implicated in the migration and invasion of human myeloid leukemia K562 cells. We performed the cell migration and invasion assays through microporous transwell membranes (transwell assay, membrane pore size of 8 μm), using serum gradient as a stimulator of cell migration and invasion (see Section 2). To determine K562 invasion, the upper chambers of transwell inserts were pre-coated with Matrigel (Corning, NY, USA) before the addition of the cells. The inhibitors of SK or IK channel activity (apamin or TRAM-34, respectively) were added to the upper and lower wells of the transwell systems installed in 4-well cell culture plates. We counted the number of migrated/invaded cells in the lower chamber after 4 h from the start of the experiment. Our data showed that apamin and TRAM-34 inhibited both the migration and invasive abilities of K562 cells. Importantly, the inhibition of cell migration/invasion was observed in the presence of 300 pM of apamin (concentration that affects only SK2 channels). This could indicate the dominant role of SK2 channels in the studied processes, as no significant differences in the effects of higher apamin concentrations (8 nM and 300 nM) were observed compared to 300 pM. A concentration of 5 μM of TRAM-34 had a more prominent effect on K562 migration compared to 1 μM, whereas both concentrations inhibited the invasion to a similar extent (Figure 4). 

### 3.5. SK or IK Channel Inhibitors Decrease Capacitative Ca^2+^ Entry in K562 Cells

**Figure 5:**
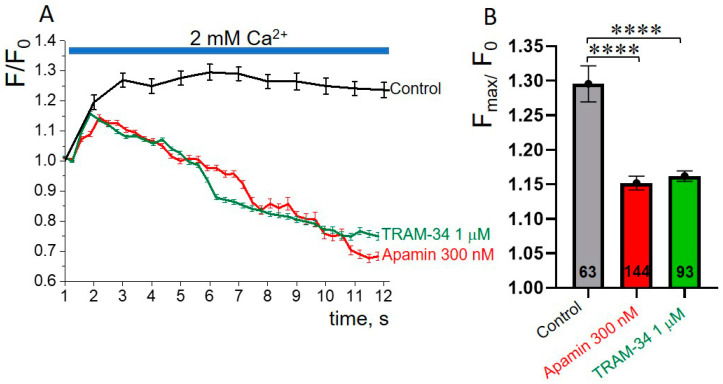
Apamin and TRAM-34 inhibit capacitative calcium entry in K562 cells. (**A**) Representative experiment demonstrating fluorescent responses of the cells (loaded with Fluo8 Ca^2+^probe) induced by addition of 2 mM Ca^2+^ in control and in the presence of apamin (300 nM) or TRAM-34 (1 µM). Each of the experiments was repeated at least 3 times. Before Ca^2+^ imaging, the cells were pre-incubated with 1 µM thapsigargin in “0 Ca^2+^” solution for 5 min to reliably deplete endoplasmic reticulum Ca^2+^ stores (see Section 2). Shown are mean fluorescence (F) intensities (± SEM) at each timepoint, normalized to the intensity (F_0_) before addition of 2 mM Ca^2+^ (in 0 Ca^2+^ solution). In this particular experiment, n = 63,144 and 93 cells were analyzed in control, in the presence of 300 nM apamin or 1 µM TRAM-34. (**B**) Maximal values (F_max_/F_0_, mean ± SEM) in 2 mM Ca^2+^ in control and in the presence of SK or IK channel inhibitors are shown. **** (*p* < 0.0001), significantly different, compared to control, two-tailed Student’s *t*-test.

One of the mechanisms that could underlie the role of SK and IK channels in the inhibition of the migrative, invasive and proliferative capabilities of K562 leukemia cells is associated with the involvement of KCa channels in the regulation of Ca^2+^ entry. Previously, the control of Ca^2+^ influx by KCa channels was shown in several types of cancer, for example, prostate and breast cancer cells [30,31]. In our experiments, we tested if SK or IK channel blockers (apamin or TRAM-34) could modulate Ca^2+^ entry in K562 cells. We performed Ca^2+^ imaging experiments in which we applied the inhibitors in Ca^2+^-containing physiological solutions to reveal any effects on capacitative Ca^2+^ entry after depletion of endoplasmic reticulum Ca^2+^ stores with the Sarco/Endoplasmic Reticulum Ca^2+^ ATPase (SERCA) inhibitor thapsigargin (TG). To reliably synchronize the cells in terms of the status of the endoplasmic reticulum Ca^2+^ stores (depleted), the cells were pre-incubated in TG-containing Ca^2+^-free solution for 5 min before the main experiment (for details, see Section 2 and Supplementary Figure S3). After this, the cells were placed in a Ca^2+^-free solution without TG and the initial values of cell fluorescence (F_0_) were established (the start of the experiment). Then, the solution was changed to 2 mM Ca^2+^ and fluorescent signals from the cells were monitored and recorded in real time. Under control conditions, the addition of 2 mM Ca^2+^ resulted in an increase in Fluo8 fluorescence, indicating an increase in [Ca^2+^]_i_. The addition of apamin (300 nM) or TRAM-34 (1 µM) to a 2 mM Ca^2+^ solution resulted in a significant decrease in Ca^2+^ responses in K562 cells, as compared to control (Figure 5A,B). Particularly, the application of apamin or TRAM-34 in 2 mM Ca^2+^ resulted in short-term (2–3 s) increase in [Ca^2+^]_i_^,^ followed by the lowering of F values below the F_0_, which indicates a decrease in [Ca^2+^]_i_ compared to the starting time point (F_0_, [Ca^2+^]_i_ in cells after TG treatment). Our data imply the involvement of SK and IK channels in the control of Ca^2+^ entry, which could (at least partially) mediate the inhibitory effects of selective SK/IK channel blockers on the pathophysiological processes in human leukemia K562 cells. 

## 4. Discussion

**Figure 6:**
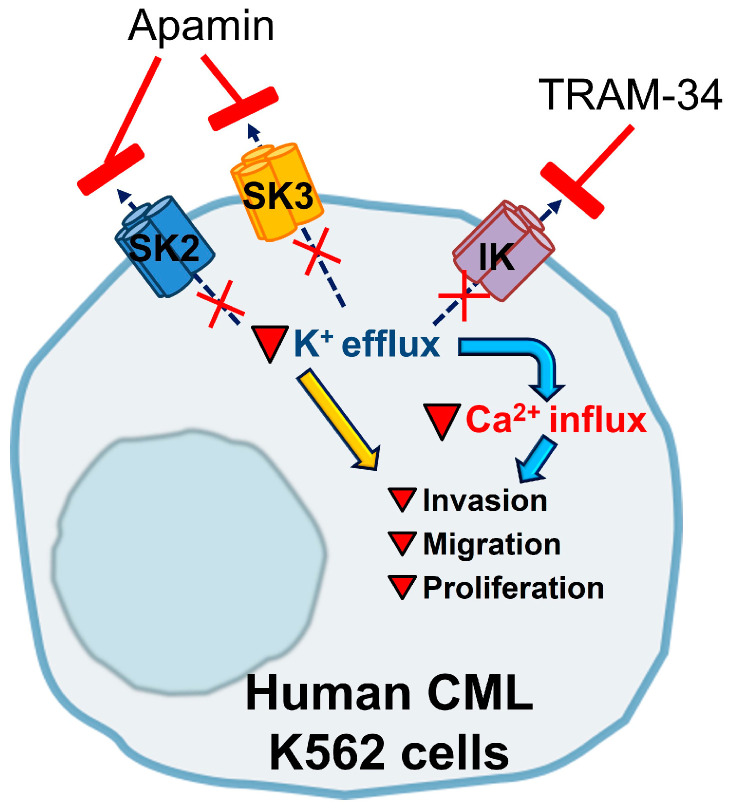
The role of KCa channels in proliferation, migration and invasion of human CML K562 cells. Functional expression of SK2, SK3 and IK channels was shown using PCR, immunofluorescence and electrophysiological assay, using selective channel inhibitors (apamin and TRAM-34). Blocking of IK- and SK-mediated K^+^ currents resulted in a decrease in Ca^2+^ influx and reduced the proliferative, invasive and migratory activities of K562 cells.

The main goal of the present study is to identify the KCa channels whose activity could be controlled by local calcium influx via mechanosensitive Ca^2+^-permeable channels in the plasma membrane of chronic myeloid leukemia K562 cells. Further, we probed to identify the role of KCa channels in the pathophysiological reactions of the cells. The results of our experiments are summarized in Figure 6. Using electrophysiological experiments with selective pharmacological channel inhibitors, we evidenced the presence of functionally active SK2, SK3 and IK channels in the human myeloid leukemia K562 cell membrane. It is of specific interest to determine why K562 cells need the functional expression of three different subtypes of KCa channels in the plasma membrane. A possible explanation is that the SK and IK channels are known to be activated by slightly different [Ca^2+^]_i_ levels, from about 100 nM for IK channels (that is, a near-basal [Ca^2+^]_i_ in most cell types), to 300–700 nM of Ca^2+^, which was evidenced to cause 50% activity in SK channels [32,33]. Thus, the presence of both SK and IK channels in the plasma membrane of the same cell allows it to react to a broader range of Ca^2+^ concentrations and to fine-tune cellular responses to the degree of Ca^2+^ increase.

We separated the activity of SK channel subtypes and showed the presence of SK2- and SK3-mediated K^+^ currents, according to their different sensitivity to apamin. We observed a sequential decrease in whole-cell currents in response to the addition of apamin in picomolar and then in nanomolar concentrations. It should be specifically noted that SK channels could co-assemble as a combination of the subtypes (i.e., form a hybrid heteromeric SK2/SK3 channel complex [22,34,35]). Such hybrid channels were reported to have intermediate sensitivity to apamin compared to SK2 and SK3 homomers and we could not exclude this situation in K562 cells. Nevertheless, the observed inhibition of SK currents both by picomolar and nanomolar ranges of apamin concentration indicates that homomeric SK2 and SK3 channels are definitely present in the plasma membrane. 

The presence of mRNA of SK2, SK3 and IK channels was confirmed by RT-PCR, whereas SK1 mRNA was not detected. Evidently, there is specific importance for the use of the combinative approach of molecular biology supplemented with electrophysiological analysis to identify the presence of functionally active ion channels in the plasma membrane. As an illustrative example, in glioma cells, the PCR analysis detected the presence of mRNA of all KCa subtypes (SK1–3, IK and BK channels) [36]. However, only pharmacological inhibitors of BK channels blocked K^+^ currents and, at the same time, SK/IK inhibitors had no effect that directly indicated the absence of functionally active SK/IK channels in the plasma membrane of glioma cells [36]. Thus, the detection of ion channel mRNA in cell lysates does not guarantee the expression of the channel proteins and a combinatorial approach is necessary to confirm the functional activity of ion channels in the plasma membrane. 

In the current work, immunofluorescent staining using specific antibodies allowed visualization of SK2, SK3 and IK channel proteins in K562 cells, whereas whole-cell patch–clamp experiments clearly evidenced the activity of functional channels in the plasma membrane. It should be noted that the fluorescent staining was clearly observed in the cytosol of the cells, indicating the localization of the channels in intracellular compartments (Figure 1D). Indeed, the presence of SK and IK channels was reported in endoplasmic reticulum, cell nuclei and mitochondria [37], as well as in vesicular transporting organelles [38]. Thus, the observed cytosolic staining could be due to the interaction of specific antibodies with intracellular SK2, SK3 and IK channel proteins. 

Our results demonstrate that blocking of SK or IK channels in K562 cells reduced their proliferative capabilities without having any significant effect on cell viability. Consistently, the inhibition of cell proliferation after blocking of IK currents was previously observed in pancreatic, prostate and colorectal cancer, as well as lung adenocarcinoma cells [30,39,40,41], as was recently reviewed by Mohr et al. [42]. In the paper by Millership et al. [43], it was documented that TRAM-34 reduced cell proliferation of human embryonic kidney (HEK293) cells transfected with recombinant human IK channels. At the same time, the overexpression of IK mutants (that are unable to conduct K^+^ or have impaired membrane trafficking) increased cell proliferation, thus indicating putative non-channel functions of IK proteins. In this case, the genetic manipulation of IK expression could lead to unpredictable results, thus making the selective pharmacological approach highly relevant to probe the channel functions in cell reactions. Importantly, TRAM-sensitive K^+^ currents were not observed in HEK293 cells, with the expression of both mutants indicating that the K^+^-selective transporting pathway formed by IK channels is not crucial for the control of cell proliferation. In further experiments, the authors showed the direct interaction of IK proteins with the ERK1/2 and JNK pathways [43]. The non-channel function of IK protein as a regulator of cell proliferation via ERK1/2 and JNK signaling pathways is a very intriguing discovery and this type of regulation could also work in the K562 leukemia cell line. Nevertheless, our data demonstrate (1) specific channel activity of IK channels in K562 cells that could be selectively inhibited by TRAM-34 in a whole-cell patch–clamp assay and (2) the significant decrease in cell proliferation in response to 5 μM TRAM-34. The identification of alternative signaling pathways that could mediate the regulation of cell proliferation by IK channels (and are not linked with their channel function) in K562 cells could be specifically addressed in future studies. 

There are some contradictory data on the role of SK channels in the proliferation of cancer cells. In mammary cancer cells, no inhibition of cell proliferation was observed in the presence of apamin [44], whereas cell migration was significantly inhibited. At the same time, the inhibition of SK channels by a glycerophospholipid, Edelfosine, in the urothelial carcinoma cell line resulted in an antiproliferative effect on the cells [45]. Also, apamin was demonstrated to slow down the growth of human melanoma IGR1 cells under hypoxic conditions [46]. In our experiments, we have evidenced an apamin-dependent decrease in K562 cell proliferation that could indicate the role of SK channels in the multiplication of leukemia cells. We did not observe any significant changes in cell viability after 24 or 48 h of incubation with apamin or TRAM-34. At the same time, treatment of the cells with 10 mM TEA slightly lowered the viability of K562 cells after 48 h. The more significant effect of 10 mM TEA on cancer cell viability was reported earlier [47]: about 50% of cervical cancer HeLa cells were dead after treatment with 10 mM TEA for 48 h. In our assay, 10 mM TEA almost completely blocked cell proliferation after 24 and 48 h of incubation, whereas a negative effect on cell viability was observed only after 48 h. The inhibition of proliferation by TEA was previously shown on C6, 9L glioma, cervical carcinoma SiHA and endometrial adenocarcinoma HEC1-A cell lines [48]. In glioma cells, treatment with TEA significantly increased the number of reactive oxygen species (ROS) and altered B-cell lymphoma protein 2/B-cell lymphoma protein-associated X (Bcl-2/Bax) balance, which further resulted in cell apoptosis [49]. In cervical carcinoma, cell cycle arrest in the Go/G1 phase due to inhibition of voltage-gated potassium (Kv) currents by 10 mM TEA was reported [50]. Since TEA could inhibit a broad spectrum of potassium channels from different families, the specific channel targets of TEA, as well as the signaling pathways affected by TEA treatment in human leukemia cells, could hardly be reliably determined. The role of K^+^ channels in the proliferation of K562 cells was demonstrated in our experiments. 

We observed that apamin or TRAM-34 inhibited both the migratory and invasive abilities of K562 cells. The effect of apamin was prominent at low concentrations (300 pM, blocking of SK2 channels), which could indicate the dominant role of SK2 channels in the studied processes. Our data confirm several studies where SK channels were shown to be involved in cell migration of tumor breast tissues [44] and invasion of urothelial carcinoma cells [45]. Also, the inhibition of cancer cell invasion and metastasis by selective IK channel inhibitors, including TRAM-34, was observed in several cancer cell lines [42]. Interestingly, the anomalous effects of TRAM-34 on pancreatic cancer cells were reported [39]. Particularly, TRAM-34 (10 μM) inhibits IK currents and stimulates migration and invasion, whereas the proliferation of pancreatic cancer cells decreases. In our experiments, the inhibition of migration and invasion of K562 cells was observed in the presence of 1 μM and 5 μM of TRAM-34, while 5 μM of TRAM-34 had a more prominent effect on cell migration. Thus, IK channels could play a complex role in cancer cell migration and the potentially unfavorable effects of IK channel inhibition should be taken into account. Most likely, the effects of KCa inhibitors could be cell-type specific and dependent on the specific signaling pathways in which the channels are integrated. 

Our Ca^2+^ measurements with the addition of selective pharmacological inhibitors of identified KCa channels (apamin and TRAM-34) demonstrate a possible mechanism which explains the suppression of K562 proliferation, migration and invasion. We observed that the inhibition of SK or IK channels resulted in a significant decrease in capacitative Ca^2+^ entry induced by the depletion of intracellular Ca^2+^ stores by TG. Both KCa channel inhibitors reduced F/F_0_ to a level below 1.0. The possible interpretation of the observed effect is that Ca^2+^, which was released from Ca^2+^ stores in response to TG, pre-activated KCa channels and then the channels, in the absence of channel inhibitors, were further activated by the Ca^2+^ entry after the addition of Ca^2+^ to extracellular solution. The addition of KCa channel inhibitors resulted in a significant reduction in K^+^ efflux, which resulted in the modulation of membrane potential, followed by changes in intracellular [Ca^2+^]_I_,_,_ which are seen as a decrease in F/F0 below 1.0. However, the interplay between KCa channel activity, Ca^2+^ release in response to TG and Ca^2+^ influx from extracellular solutions could be more complex. 

Our data showed that the activity of SK and IK channels can critically regulate Ca^2+^ entry and, in turn, modulate various cell responses. A similar mechanism was previously shown in several studies and functional partners (that is, various Ca^2+^-permeable ion channels) that mediate Ca^2+^ influx have been identified for SK and IK channels. Particularly, Bi et al. demonstrated that inhibition of IK channels leads to suppression of Ca^2+^ influx and, consequently, slows down the proliferation of vascular smooth muscle cells [51]. In renal collecting duct cells, SK and IK channels regulate the activity of TRPV4 channels that stimulate Ca^2+^ influx [52]. Also, IK channels can regulate Ca^2+^ influx by functional cooperation with TRPC1 channels in the MCF-7 cell line [31] and the inhibition of IK channels leads to the decrease in MCF-7 proliferation caused by Ca^2+^ influx suppression. Lallet-Daher et al. showed that IK channels control TRPV6-mediated Ca^2+^ entry in LNCaP and PC-3 prostate cancer and inhibition of IK channels also suppressed the proliferation of cancer cells [30]. The particular Ca^2+^ channels whose activity may be influenced by the inhibition of SK or IK channels in K562 cells could be identified in further studies. 

KCa channels can be considered an attractive target for the treatment of various diseases and are among FDA-approved drug targets (see KCNN1-4, https://www.proteinatlas.org/search/protein_class%3AFDA+approved+drug+targets/10, accessed on 27 April 2023). For instance, IK channels appear to play a more significant role in the development of different pathological conditions compared to normal physiology [53]. The KCa3.1 blocker clotrimazole has been clinically tested for the treatment of rheumatoid arthritis [54] and RBC dehydration in sickle cell disease [55]. Although clotrimazole was effective in treatment, it had many side effects that limited its use. The discovery of the clotrimazole analogs TRAM-34 and senicapoc [56], which are more selective for the KCa3.1 channel, may have clinical significance. The intravenous administration of TRAM-34 (0.5 mg/kg; 29 μM) had no toxic effect on CF-1BR mice at a concentration 500–100 times higher than the dose that is sufficient to block IK channels [57]. Moreover, this concentration of TRAM-34 has been shown to have several protective effects on different cells and tissues: immunosuppressive effect, lymphocyte mitogenesis [57], reversal of renal damage in mice [58] and reversal of infarction and neurological deficit in ischemia/reperfusion stroke rat model [59]. Therefore, the administration of high doses of TRAM-34 to mice and rats for a week had no toxic effect and only minor side effects. Apamin was shown to be a potential pharmacological agent to treat apoptosis, fibrosis and the diseases of the central nervous system (recently reviewed by Gu et al. [60]). To summarize, the IK and SK channel blockers TRAM-34 and apamin were tested and showed several positive modulatory effects on various diseases. It can be assumed that KCa channels could also be targeted to treat several types of human cancer. 

In our experiments, we observed a decrease in CML K562 cell migration and invasion in the presence of selective blockers of SK or IK channels. K562 cells are a specific cell line with molecular pathways that are not present in other types of leukemia. K562 cells are extremely resistant to apoptosis irrespective of the pharmacological compound used for its induction and this feature was shown to be due to the expression of the Bcr-Abl protein [61]. The tyrosine kinase inhibitors are successfully used as a target therapy for the treatment of CML [62,63]. The possible positive combinatory effects of tyrosine kinase inhibitors and SK/IK channel blockers on the pathophysiological reactions of K562 cells could be a specific goal for future studies. 

It should be noted that different leukemia cells (or other blood cancers) are expected to have variable patterns of KCa channel expression and regulation, i.e., the presence of specific KCa subtypes, the number of functionally active KCa channels on the plasma membrane or Ca^2+^ sources that could regulate KCa activity in the cells. Recently, we demonstrated the functional expression of BK channels in the Burkitt’s lymphoma Raji cell line and SK-/IK-like currents were also present, whereas no BK currents were observed in K562 cells. Importantly, both BK and SK/IK channel activity in Raji cells was dependent on calcium influx via mechanosensitive ion channels [64]. The Jurkat cell line, a commonly used experimental model of T cell leukemia, expresses functionally active SK2 channels but no IK currents, whereas IK-mediated currents were observed in other leukemic MOLT-3 cells [12]. Thus, our results are rather limited to a specific type of leukemia and our data imply that SK/IK channel inhibitors could be used to slow down the proliferation and spreading of CML K562 cells that express functionally active SK2, SK3 and IK channels in the plasma membrane.

## Figures and Tables

**Table 1:** Primer sequences for *KCNN1-4* genes used in this study.

Gene (KCa Type)	Forward Primer Reverse Primer	Predicted Product Size, bp
*hKCNN1* (KCa2.1, SK1)	3′-AGA ACA GCA AGA CAT ATC CG-5′ 5′-ATT GTA GCT GTG GCT GTT CA-3′	282
*hKCNN2* (KCa2.2, SK2)	3′-CTT ATC AGT CTC TCC ACG ATC-5′ 5′-TAC AGT TCC TGG GCA TAT AG-3′	423
*hKCNN3* (KCa2.3, SK3)	3′-CGA CTG AGT GAC TAT GCT C-5′ 5′-GTG GAC AGA CTG ATA AGG C-3′	137
*hKCNN4* (KCa3.1, IK)	5′-GGC CAA GCT TTA CAT GAA C-3′ 3′-ATC ATG AAG TTG TGC ACG TG-5′	325

## Data Availability

The data presented in this study are available within the article and upon request.

## Supplementary Materials

The following supporting information can be downloaded at: https://www.mdpi.com/article/10.3390/membranes13060583/s1, Figure S1: The action of TEA and pCa8 on whole-cell currents in K562 cells. Figure S2: Original gels showing the presence of SK2, SK3 and IK mRNA and the absence of SK1 mRNA; Figure S3: Dynamics of TG-induced Ca^2+^ store depletion in K562 cells. Supplementary Table 1: Summary of *p*-values for K562 proliferation and cell viability experiments.
