# Supplementary material for: Role of Calcium-Activated Potassium Channels in Proliferation, Migration and Invasion of Human Chronic Myeloid Leukemia K562 Cells
Source: Membranes (Basel). 2023 Jun 4;13(6):583. doi: 10.3390/membranes13060583 (PMC10303497; doi:10.3390/membranes13060583)
Supplement: Supplementary file 1 [file membranes-13-00583-s001.zip › Supplementary Figures.pdf]

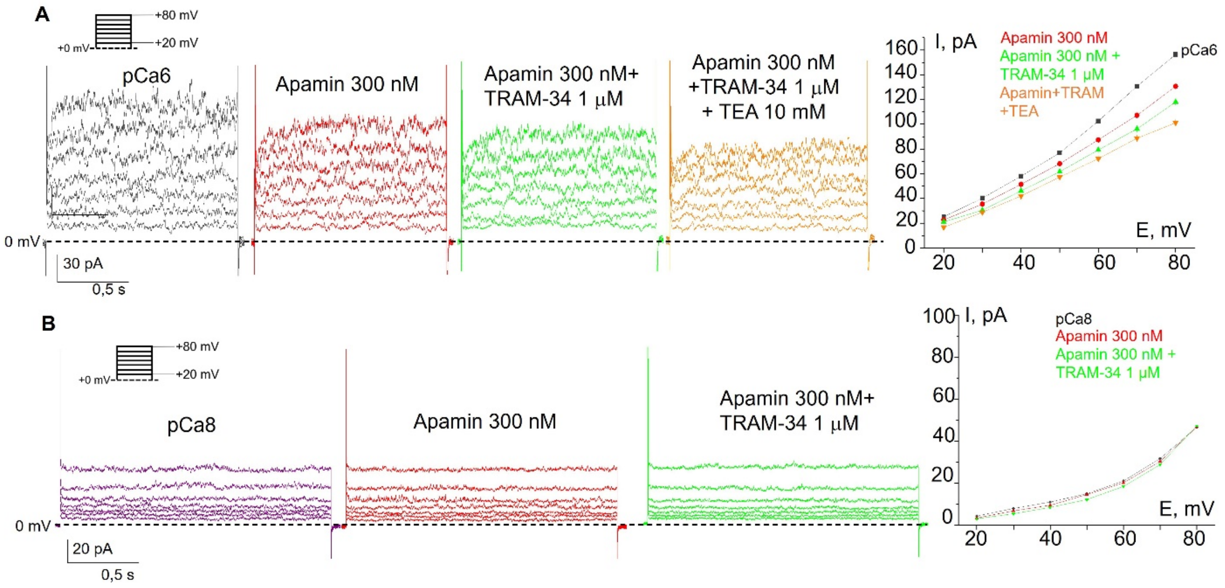

Supplementary Figure S1. The action of TEA and pCa8 on whole-cell currents in K562 cells. Shown are representative experiments demonstrating (A) the effect of 10 mM TEA, a non-specific K channel blocker, on whole-cell currents in the plasma membrane of K562 cells. Note a further decrease of outward currents after the application of TEA. (B). Whole-cell currents in the presence of low Ca^{2+} concentration in recording pipette (mM):140 KAspartate, 5 NaCl, 2 EGTA/KOH,1 $MgCl_2$, 20 HEPES/TrisOH with necessary amount of $CaCl_2$ to establish free ionized calcium concentration $[Ca^{2+}]_i$ at 10 nM (pCa8). No significant effects of apamin and TRAM-34 on whole-cell currents are observed. Also note the smaller amplitudes of currents in pCa8, comparing to pCa6 (panel A and Figure 1).

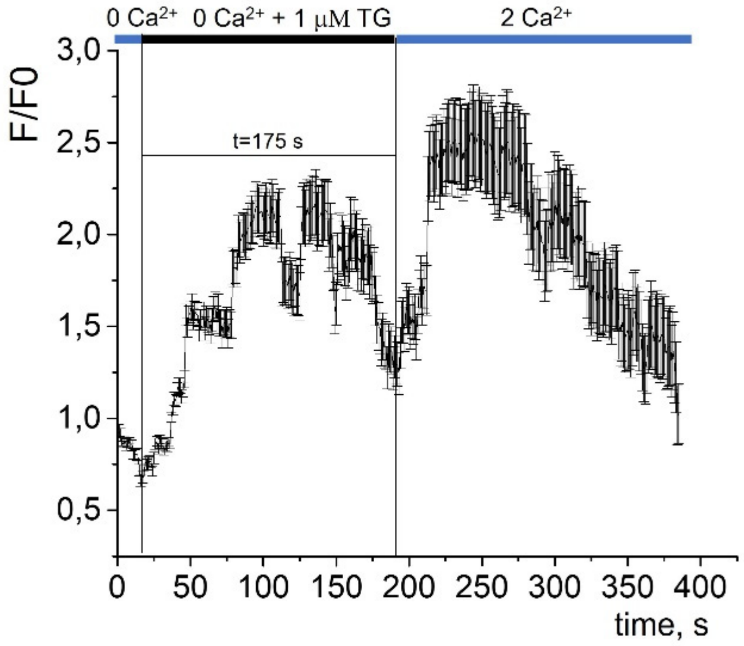

Supplementary Figure S3. Dynamics of TG-induced Ca^{2+} store depletion in K562 cells. Ca^{2+} responses of K562 cells to the application of 1 μM of thapsigargin (TG) in Ca^{2+}-free solution (0 Ca^{2+}). The addition of 2 mM Ca^{2+} further increase further increase Fluo8 fluorescence and, accordingly, Ca^{2+} levels in the cells.

Shown are mean fluorescence (F) intensities (± SEM) at each timepoint normalized to the intensity (F_0) at the beginning of the experiment (in 0 Ca^{2+} solution, n=28 cells used for the analysis). Note that TG-induced Ca^{2+} response lasts about 3 minutes (175 s).
