# Supplementary material for: Role of Calcium-Activated Potassium Channels in Proliferation, Migration and Invasion of Human Chronic Myeloid Leukemia K562 Cells
Source: Membranes (Basel). 2023 Jun 4;13(6):583. doi: 10.3390/membranes13060583 (PMC10303497; doi:10.3390/membranes13060583)
Supplement: Supplementary file 1 [file membranes-13-00583-s001.zip › Supplementary Table 1.pdf]

	Proliferation		Cell viability			Proliferation	Cell viability
	24 h	48 h	24 h	48 h		p-value	p-value
Control vs Apamin 300 nM	0.0188	0.0003	0.8909	0.1913	Control 24h vs Control 48h	<0.0001	**0.0376** (93.64%/96.30%)
Control vs TRAM-34 5 mkM	**0.0844** (ns)	0.0017	0.2813	0.1640	Apamin 24h vs Apamin 48h	0.0003	**0.0254** (93.87%/98.01%)
Control vs TEA 10 mM	<0.0001	<0.0001	0.4003	<0.0001	TRAM-34 24h vs TRAM-34 48 h	<0.0001	**0.0204** (95.46%/97.83%)
					TEA 24 h vs TEA 48 h	0.302 (ns)	**0.0463** (95.12%/88.97%)

Supplementary Table 1. Summary of p-values for K562 proliferation and cell viability experiments (see Figure 3). Significant differences in cell viability when comparing each experimental condition at 24h and 48h could be observed. However, the number of viable cells after 48 h is higher than at 24 h, except TEA. These data further supply the observed lack of effect of apamin and TRAM-34 on K562 cell viability. One-way ANOVA with Dunnett's multiple comparisons.
