# Supplementary figures and images for: Role of Calcium-Activated Potassium Channels in Proliferation, Migration and Invasion of Human Chronic Myeloid Leukemia K562 Cells
Source: Membranes (Basel). 2023 Jun 4;13(6):583. doi: 10.3390/membranes13060583 (PMC10303497; doi:10.3390/membranes13060583)

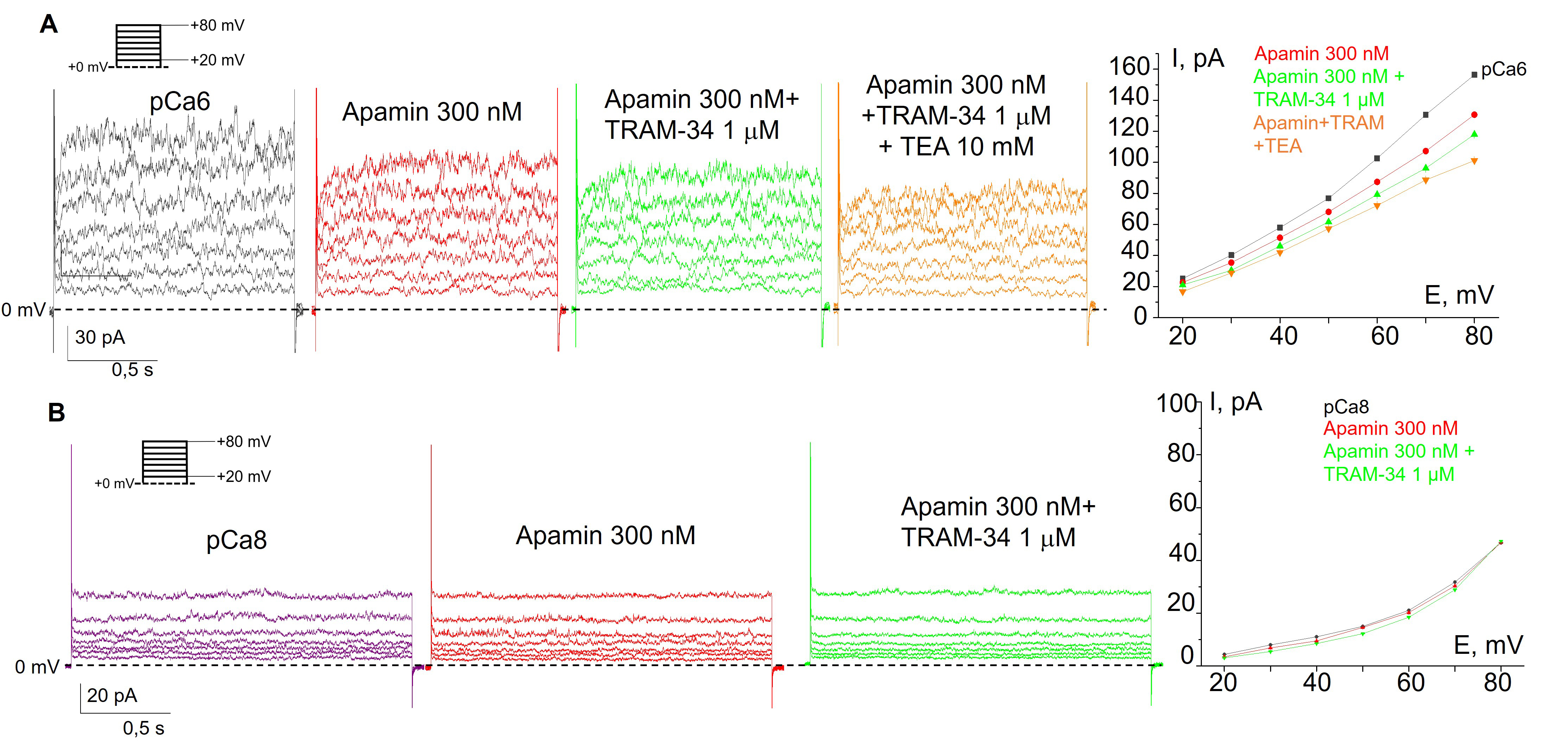

Supplement: Supplementary file 1 [file membranes-13-00583-s001.zip › Supplementary figure S1.jpg]

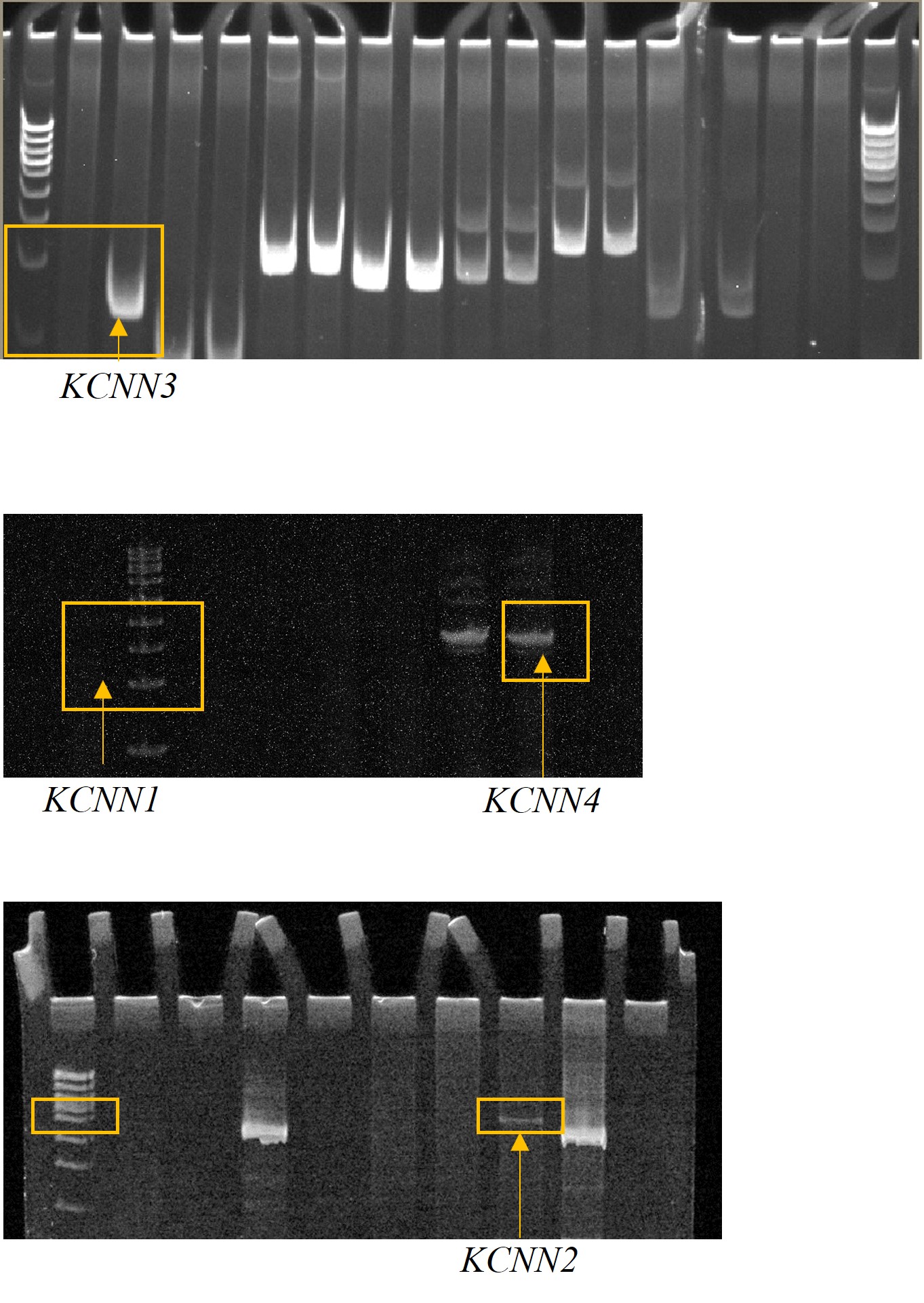

Supplement: Supplementary file 1 [file membranes-13-00583-s001.zip › Supplementary Figure S2.jpg]

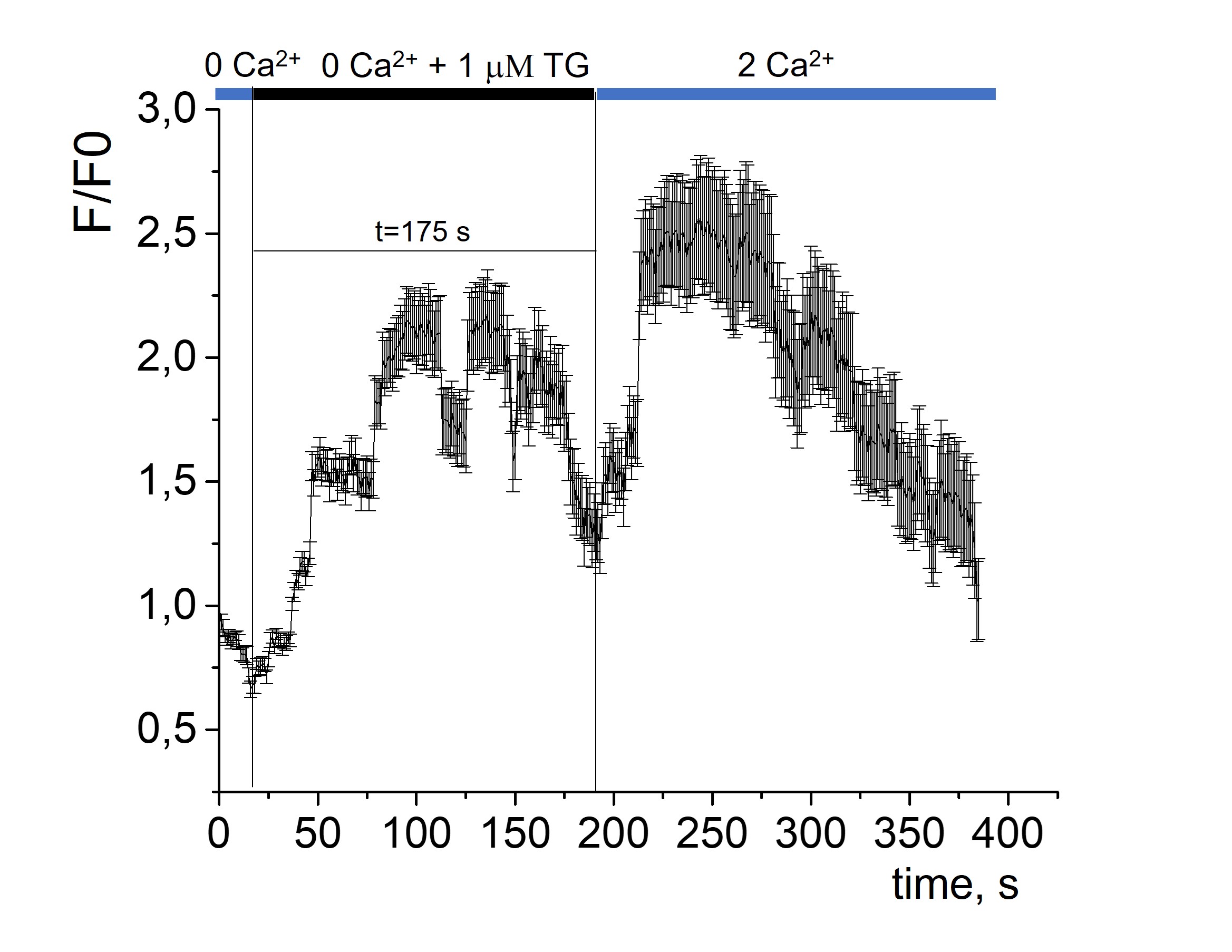

Supplement: Supplementary file 1 [file membranes-13-00583-s001.zip › Supplementary Figure S3.jpg]
